# Malignant acanthosis nigricans: an early diagnostic clue for gastric adenocarcinoma

**DOI:** 10.1186/s12957-017-1274-5

**Published:** 2017-11-25

**Authors:** Qian Yu, Xiu-Li Li, Guo Ji, Yao Wang, Yu Gong, Hui Xu, Yu-Ling Shi

**Affiliations:** 1 Department of Dermatology, Shanghai Tenth People’s Hospital9, Tongji University School of Medicine, No.301, Middle Yanchang Road, Shanghai, 200072 China; 2 Department of Pathology, Shanghai Tenth People’s Hospital, Tongji University School of Medicine, Shanghai, 200072 China

**Keywords:** Malignant acanthosis nigricans, Hyperkeratosis, Papillomatosis, Gastric adenocarcinoma

## Abstract

**Background:**

Malignant acanthosis nigricans (MAN), characterized by the presence of a hyperpigmented, velvety cutaneous thickening, is recognized as a cutaneous sign of internal malignancy. Few MAN has been reported in the Asian race ever before.

**Case presentation:**

Here, we report a rare case of MAN with severe mucosa and soles and extraordinary facial involvement in the Asian race. A 74-year-old man presented with hyperkeratotic eruption for 7 months. Physical examination revealed hyperkeratotic plaques on the face, dorsal skin of fingers and heels, and papillomatosis of buccal mucosa. Biopsy findings from skin lesion revealed hyperkeratosis, papillomatosis, and hyperpigmentation of the basal layer. The endoscopic ultrasound with biopsy of the gastric tissue revealed gastric cardia tubular adenocarcinoma. The patient was diagnosed with MAN associated with gastric adenocarcinoma, immediately following tumor resection and lymphadenectomy. A slight improvement was seen in the skin condition but died of cancer cachexia 3 months later.

**Conclusions:**

We report our typical patient to highlight the importance of MAN, which was an early clue to the discovery of gastric adenocarcinoma.

## Background

Malignant acanthosis nigricans (MAN), characterized by the presence of hyperpigmented, hyperkeratosis, and cutaneous thickening of the skin or mucous membranes, is recognized as a cutaneous sign of internal malignancy [1]. The disease mostly occurs in the elderly without gender differences, and gastric carcinoma is the most common with the incidence of 55–61% in patients with MAN, followed by pancreatic cancer, gynecological malignancies, and lung carcinoma [2]. As a paraneoplastic type of dermatosis, MAN frequently has more severe skin and mucous lesions characterized with sharp deterioration and rapid spread than those in the benign form.

MAN can occur simultaneously, before or after the onset of internal malignancy [3]. Therefore, it needs to be recognized by dermatologists to make an early diagnosis of MAN and to improve the prognosis related to the underlying malignancy. To our knowledge, few MAN has been reported in the Asian race ever before. Here, we report a rare case of MAN with severe mucosa and soles and extraordinary facial involvement in the Asian race, which was the first sign presented prior to the discovery of gastric adenocarcinoma.

## Case presentation

A 74-year-old man was admitted to our hospital with symmetric hyperkeratotic skin changes for a 7-month duration. Clinical manifestation showed hyperkeratotic plaques, first affecting the oral mucosa, face, and fingers then extending to palms, soles, and trunk. Meanwhile, the patient also complained of heartburn and felt bloated after meals during the last 2 months. The patient had no other obvious medical history. He denied any history of similar diseases in his family. The patient was a common farmer around the countryside and often planted in farmland under the sun. He lived in the southeast of China. He was married and reported no drug abuse history. He drank alcohol occasionally and was a former smoker.

**Fig. 1:**
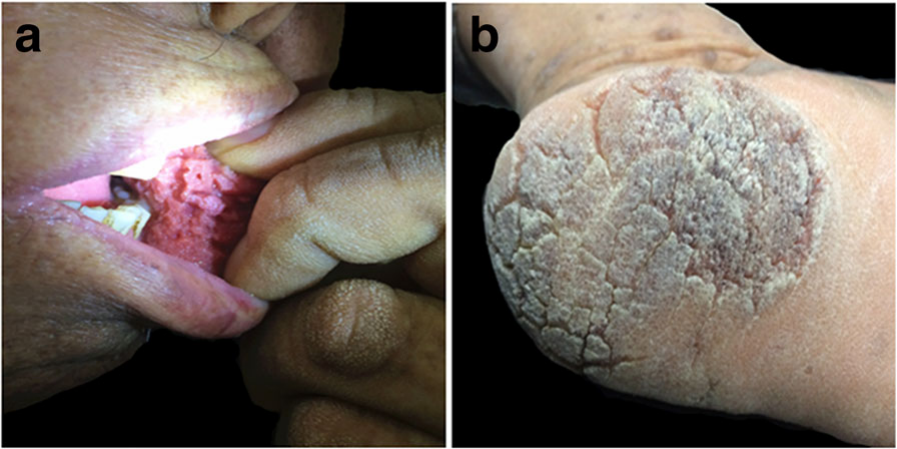
The distribution of lesions. Oral florid papillomatosis was observed on the buccal mucosa (**a**), and hyperkeratotic plaques were observed on soles (**b**)

On physical examination, hyperkeratotic plaques were observed in the oral mucosa, face, fingers, palms, soles, and trunk (Fig. 1). No lesions were detected in the wrinkles of the neck, armpits, groin, and other parts of the body. The other physical and neurological examinations were unremarkable. Serum carcinoembryonic antigen (CEA) was 100.30 μg/L (normal range 0 to 5), and carbohydrate antigen 199 (CA199) was 250.98 u/mL (normal range 0 to 40). Other laboratory findings were within normal range, and no other clinically significant findings were noted.

**Fig. 2:**
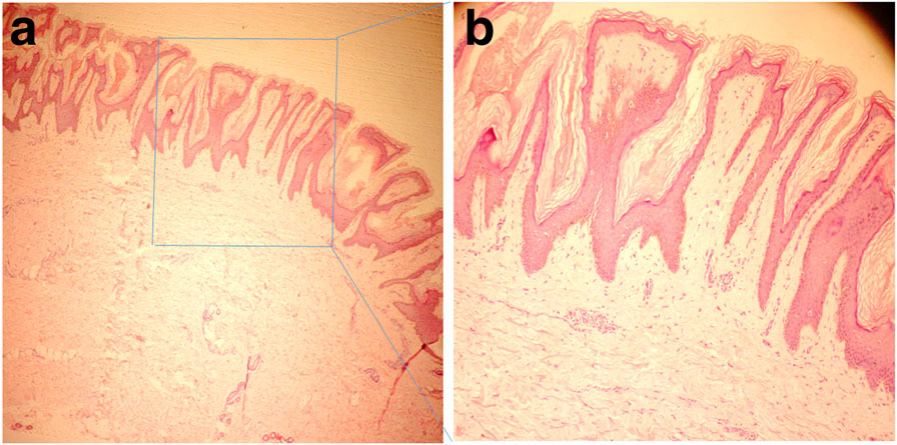
Histopathologic examinations of the skin lesion. A biopsy of the skin lesion revealed epidermal hyperkeratosis, papillomatosis, and hyperpigmentation of the basal layer. In addition, some dermal papillae were projected upward in the form of finger-like projections (**a** HE × 40; **b** HE × 100)

**Fig. 3:**
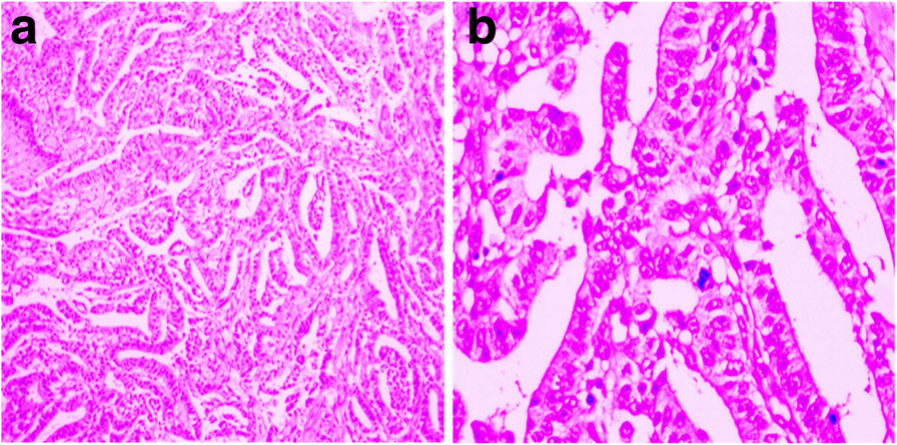
Histopathologic examinations of the gastric tissue. A biopsy of the gastric tissue showed dilated branching tubules of varying diameter (HE × 200) (**a**); the malignant glands invade the fibrous stroma, and the degree of nuclear atypia varies from middle to high grade (HE × 400) (**b**)

A biopsy of skin lesion revealed hyperkeratosis, papillomatosis, and hyperpigmentation of the basal layer, and some dermal papillae were projected upward in the form of finger-like projections (Fig. 2). Furthermore, an esophagogastroduodenoscopy was performed. A histopathological examination of the gastric mucosa showed dilated branching tubules of varying diameter and presence of acinar structure. In addition, the malignant glands invaded fibrous stroma, and individual neoplastic cells were columnar or cuboidal, with the varying degree of nuclear atypia from middle to high grade (Fig. 3). An endoscopic ultrasound was performed to determine the depth of invasion and lymph node metastasis, which led to the diagnosis of gastric cardia tubular adenocarcinoma and TNM classification (T4N2M0). The patient was detected with lymph node metastasis in the abdominal cavity by a computed tomography scan.

The diagnosis of MAN associated with gastric adenocarcinoma (T4N2M0) was given. Palliative surgery was recommended to relieve symptoms and improve life quality of the patient. Then the patient was transferred to the department of gastrointestinal surgery for tumor resection and lymphadenectomy. A subsequent slight improvement was seen in the skin condition. Unfortunately, the patient died of cancer cachexia 3 months later.

## Discussion

MAN is considered as a rare paraneoplastic skin syndrome. The pathogenesis of MAN remains unclear. MAN was detected with enhanced expression of fibroblast growth factor receptor [4]. In addition, the activation of epidermal growth factor receptor/ERK signaling was correlated with suppressed differentiation in MAN [5]. Therefore, hyperkeratotic plaques may result from autoimmune antibodies and inflammatory factors that are directed against underlying tumors, and a secondary cross-reaction with the epidermis and basement membrane of the skin may induce the occurrence of the disease [6].

Clinical manifestations of MAN are characterized by pruritic, hyperkeratotic, and hyperpigmented plaques with a subsequent formation of velvety papillomas in the involved areas, and tripe palms syndrome may be seen [7]. Lesions are frequently involved in the skin wrinkles of the neck, armpits, groin, and another axillary region of the body. However, the velvety hyperkeratotic lesions can be located on the dorsum of the hands and feet in the form of a variant of acanthosis nigricans called as acral type acanthosis nigricans, which has been reported in dark-skinned people [1]. The characteristic of tripe palms is the velvety thickening of the palms, with the exaggeration of normal skin markings [3]. Another important clinical characteristic of MAN is an improvement of the lesions that occur after tumor resection and recur when the tumor returns.

Diagnosis of MAN should be analyzed by taking a medical history, clinical symptoms and signs, and laboratory examination into consideration. A biopsy is generally helpful in the diagnosis of MAN, but its histological features are not specific. It commonly includes hyperkeratosis, papillomatosis, and hyperpigmentation of the basal layer, and some dermal papillae were projected upward in the form of finger-like projections [8]. Differential diagnoses include acrokeratosis paraneoplastica, psoriasis, tinea, eczemas, syphilis, and acquired palmoplantar keratoderma.

Treatments of MAN emphasize the primary diseases, and it is usually resistant to conventional treatments, such as coal tar, salicylic acid, vitamin D analogs, and corticoids. Systemic treatments with etretinate, corticoids, and antibiotics also showed inconsistent results. No significant clinical effects were observed in skin-directed therapy, and skin lesions can be alleviated by treating the underlying malignant condition. It has been reported that 90–95% of cases experience an improvement of the skin lesions following treatment of the primary tumors, and clinical effect could be observed within months of cancer treatment [9].

Prognosis of MAN associated with internal malignancies is poor, though skin manifestations recover after therapy in quite a few patients [7]. Tumor resection and lymphadenectomy were performed immediately in some patients; unfortunately, because of the further finding, most of them died of cancer cachexia. The main reasons were as follows. First, some patients did not pay attention to their slight skin symptoms in the early stage of an underlying malignancy and did not see a doctor in time. Second, though some patients’ clinical findings were typical, few cases associated with internal malignancies have been seen in clinical practice. Then, quite a few patients were misdiagnosed as psoriasis or another skin disease. Therefore, cancer screening should be performed in patients with hyperkeratotic papules and plaques, and early recognition of MAN may improve the prognosis of such patients.

In our study, though gastric cardia tubular adenocarcinoma is a common malignant tumor in the clinic, its early symptoms are mild or not obvious because of the special site of cardia cancer. While other MAN cases, with gastric cancer occurs in the lesser curvature and the anterior and posterior walls of the stomach, usually have typical clinical symptoms of stomach pain, nausea, and vomiting, which may prompt the patients to examine their stomach in time. However, quite a few patients with cardia cancer are in the middle and late stage when they visit a doctor, and the surgical treatment effect is usually poor. In our study, skin symptoms of the patient have been maintained for 7 months before he visited a doctor. If the patient visits the Department of Dermatology early in the discovery of skin symptoms, the tumor might be early detected, then radical surgical treatment might further prolong the survival time. Therefore, MAN should be considered as an “early” clue to the discovery of gastric adenocarcinoma.

## Conclusions

In conclusion, we present here a rare case in order to improve the identification of MAN, which could be the first sign of the early diagnosis of gastric adenocarcinoma. Furthermore, it is important for the dermatologists to recognize it in favor of early diagnosis and treatment of specific internal malignancies.

## Abbreviations

CA199: Carbohydrate antigen 199
CEA: Carcinoembryonic antigen
MAN: Malignant acanthosis nigricans

## References

[1] Lee SS, Jung NJ, Im M, Lee Y, Seo YJ, Lee JH (2011). Acral-type malignant acanthosis nigricans associated with gastric adenocarcinoma. Ann Dermatol.

[2] Kubicka-Wolkowska J, Debska-Szmich S, Lisik-Habib M, Noweta M, Potemski P (2014). Malignant acanthosis nigricans associated with prostate cancer: a case report. BMC Urol.

[3] McGinness J, Greer K (2006). Malignant acanthosis nigricans and tripe palms associated with pancreatic adenocarcinoma. Cutis.

[4] Hida Y, Kubo Y, Nishio Y, Murakami S, Fukumoto D, Sayama K, Hashimoto K, Arase S (2009). Malignant acanthosis nigricans with enhanced expression of fibroblast growth factor receptor 3. Acta Derm Venereol.

[5] Haase I, Hunzelmann N (2002). Activation of epidermal growth factor receptor/ERK signaling correlates with suppressed differentiation in malignant acanthosis nigricans. J Invest Dermatol.

[6] Ramos-E-Silva M, Carvalho JC, Carneiro SC (2011). Cutaneous paraneoplasia. Clin Dermatol.

[7] Kebria MM, Belinson J, Kim R, Mekhail TM (2006). Malignant acanthosis nigricans, tripe palms and the sign of Leser-Tre’lat, a hint to the diagnosis of early stage ovarian cancer: a case report and review of the literature. Gynecol Oncol.

[8] Bazex A, Griffiths A (1980). Acrokeratosis paraneoplastica—a new cutaneous marker of malignancy. Br J Dermatol.

[9] Cabanillas M, Perez-Perez L, Sanchez-Aguilar D, Fernandez-Redondo V, Toribio J (2006). Acrokeratosis paraneoplastica with bullous lesions associated with esophageal squamous cell carcinoma. Actas Dermosifiliogr.

